# Neutralizing antibodies for SARS-CoV-2 in stray animals from Rio de Janeiro, Brazil

**DOI:** 10.1371/journal.pone.0248578

**Published:** 2021-03-25

**Authors:** Helver Gonçalves Dias, Maria Eduarda Barreto Resck, Gabriela Cardoso Caldas, Alessandro Fioretti Resck, Natália Valente da Silva, Aline Marcele Vieira dos Santos, Thiago das Chagas Sousa, Maria Halina Ogrzewalska, Marilda Mendonça Siqueira, Alex Pauvolid-Corrêa, Flavia Barreto dos Santos

**Affiliations:** 1 Laboratório de Imunologia Viral, Instituto Oswaldo Cruz, Fiocruz, Rio de Janeiro, Brasil; 2 Laboratório de Mosquitos Transmissores de Hematozoários, Instituto Oswaldo Cruz, Fiocruz, Rio de Janeiro, Brasil; 3 Laboratório de Morfologia e Morfogênese Viral, Instituto Oswaldo Cruz, Fiocruz, Rio de Janeiro, Brasil; 4 Clínica Veterinária Animal Help, Rio de Janeiro, Brasil; 5 Laboratório de Vírus Respiratórios e Sarampo, Instituto Oswaldo Cruz, Fiocruz, Rio de Janeiro, Brasil; 6 Department of Veterinary Integrative Biosciences, Texas A&M University, College Station, Texas, United States of America; University of Lincoln, UNITED KINGDOM

## Abstract

The epidemic of coronavirus disease 2019 (COVID-19), caused by a novel *Betacoronavirus* named severe acute respiratory syndrome coronavirus 2 (SARS-CoV-2) became a public health emergency worldwide. Few reports indicate that owned pets from households with at least one human resident that was diagnosed with COVID-19 can be infected by SARS-CoV-2. However, the exposure to SARS-CoV-2 of pets from households with no COVID-19 cases or stray animals remains less assessed. Using real-time reverse transcriptase polymerase chain reaction (RT-PCR) and plaque reduction neutralization test (PRNT_90_), we investigated the infection and previous exposure of dogs and cats to SARS-CoV-2 during the ongoing COVID-19 epidemic in Rio de Janeiro, Brazil. From June to August 2020, 96 animals were sampled, including 49 cats (40 owned and 9 stray) and 47 dogs (42 owned and 5 stray). Regarding owned pets, 75.6% (62/82) belonged to households with no COVID-19 cases. Samples included serum, and rectal and oropharyngeal swabs. All swabs were negative for SARS-CoV-2 RNA, but serum samples of a stray cat and a stray dog presented neutralizing antibodies for SARS-CoV-2, with PRNT_90_ titer of 80 and 40, respectively. Serological data presented here suggest that not only owned pets from households with COVID19 cases, but also stray animals are being exposed to SARS-CoV-2 during the COVID-19 pandemic.

## Introduction

The novel *Severe acute respiratory syndrome coronavirus 2* (SARS‐CoV‐2) that belongs to the *Betacoronavirus* genus rapidly spread worldwide causing an unprecedented pandemic of coronavirus disease (COVID-19). Patients usually have clinical symptoms such as fever, cough, difficulty breathing, loss of taste or smell, headache and pneumonia, and also others clinical nonrespiratory manifestations, which can progress to severe clinical presentations and death [1, 2].

Phylogenetic analyzes showed that SARS-CoV-2 has a possible zoonotic origin, with species of bat as reservoirs [1, 3, 4]. Regarding domestic animals, it has been experimentally shown that cats are not only susceptible to SARS-CoV-2 infection, but they also have the capability to transmit the virus to others co-housed cats [5–7]. Furthermore, cats that were experimentally infected by SARS-CoV-2 mounted an effective immune response [5]. On the other hand, dogs seem to be less susceptible to SARS-CoV-2 infection presenting lower seroprevalence and limited capacity to transmit the virus [5, 8].

Since the beginning of the pandemic, different countries have reported the detection of RNA and/or specific antibodies for SARS-CoV-2 in dogs and felines [9–16]. The large majority of these studies have tested pets from households with at least one human case of COVID-19. The exposure of animals from households with no confirmed COVID-19 cases, or the exposure of stray animals to SARS-CoV-2 is being less investigated.

Companion animals, especially dogs and cats, are in close contact with humans and inhabit the same environment. Because of that, they may be highly exposed to human pathogens. It is estimated there are around 78.1 million dogs and cats as pets in Brazil. Of those, about 5% are animals in vulnerable conditions [17]. The large number of stray animals represents an important concern in the context of public health and animal welfare [9, 16].

The Brazilian Ministry of Health notified the first human confirmed case of COVID-19 in the country on February 26, 2020. By late October, about 5.5 million cases and more than 159 thousand deaths had been reported in the country [18]. Brazil has the most cases and deaths in Latin America and those numbers are probably underestimated. The Southeast Region is the most populated area of the country and concentrates the largest number of COVID-19 cases and where São Paulo and Rio de Janeiro are the most affected cities [19]. Even with the largest number of COVID-19 cases in South America, the exposure of companion animals to SARS-CoV-2 in Brazil has been poorly explored [20].

In the present study, we investigated the presence of SARS-CoV-2 RNA in oropharyngeal and rectal swabs by real-time RT-PCR, and the presence of neutralizing antibodies specific to SARS-CoV-2 in sera by plaque reduction neutralization test (PRNT_90_) in cats and dogs from Rio de Janeiro, during the ongoing COVID-19 epidemic.

## Materials and methods

### Ethical statement

This study was approved by the Oswaldo Cruz Institute’s Animal Care and Use Committee (protocol number 013/2020) in compliance with the requirements of Brazilian Law 11794/2008, and additional approvals from veterinary clinics were specific to each partner.

### Sampling

From June to August 2020, a total of 96 animals, including 49 cats (*Felis catus*) and 47 dogs (*Canis lupus familiaris*), were sampled in the city of Rio de Janeiro, Brazil. From those, 85.4% (82/96) were owned pets and 14.6% (14/96) were stray animals. Among owned animals, 75.6% (62/82) were from a household with no history of COVID-19 case and 24.4% (20/82) from a household with history of a human COVID-19 case. Convenience sampling was performed at two veterinary clinics located in the North and West zones of the city. Blood samples as well as oropharyngeal and rectal swabs were collected from each animal. After initial medical evaluation, animals were eligible for research and the owners were invited to participate. It was not an inclusion criterium that the owner had been diagnosed with COVID-19. Animals older than 14 years, less than 4 months, or pregnant were not included in the study. After acceptance, the owners underwent a quick interview with questions related to the animal’s general health status and the socio-environmental factors of the dwelling. The stray animals included in the research were captured by non-governmental organizations and assisted at both veterinary clinics.

The oropharyngeal and rectal swabs were collected and placed in tubes containing viral transport medium (VTM). VTM was prepared with 2% fetal bovine serum (FBS) (Gibco—10270106), antibiotics (100 μg/mL for gentamicin and 0.5 μg/mL for amphotericin B) and Hanks Balanced Salt Solution [21]. Blood collection was performed through femoral venipuncture in tubes with no anticoagulants. Serum was separated from blood samples by centrifugation, and then stored at -70°C freezers.

### Laboratory testing

#### Real-time RT-PCR for SARS-CoV-2

RNA extraction of VTM samples from oropharyngeal and rectal swabs was performed using the ZR-Viral RNA kit (Zymo Research, Irvine, USA) according to the manufacturer’s instructions. RNA samples were then tested for SARS-CoV-2 by two commercial kits, the Allplex^™^ 2019-nCoV Assay (Seegene Inc., Taewon, Republic of Korea) that targets the envelope (E), polymerase (RdRP) and nucleocapsid (N) genes, and the Biomanguinhos RT-qPCR kit (FIOCRUZ, Brazil) that targets the E gene, according to manufacturer’s instructions.

#### Plaque reduction neutralization test (PRNT_90_)

The PRNT is a highly specific serologic test and can be carried out on samples to confirm the presence of neutralizing antibodies to SARS-CoV-2 [22, 23]. All serum samples were heat-inactivated and tested by for their ability to neutralize plaque formation by SARS-CoV-2. The assay was performed in a Multi-user Research Facility of Biosafety Level 3 Platform of Instituto Oswaldo Cruz/FIOCRUZ. The reference SARS-CoV-2 used for PRNT_90_ was isolated from a patient from Rio de Janeiro and belongs to lineage B.1. Full genome sequence is deposited at GISAID (EPI_ISL_414045).

Vero cells (ATCC, CCL 81) were maintained in 5% CO_2_ atmosphere at 37°C in 199 Medium supplemented with 10% FBS (Gibco—10270106), buffered with sodium bicarbonate (0,075g/mL), 100 U/mL penicillin, 0,1 mg/mL streptomycin, 40 ug/mL gentamicin and 0.025 ug/mL amphotericin B. Briefly, inactivated aliquots were screened at a dilution of 1:10 in two-day old Vero CCL-81 cells seeded in 6-well plates. Those that neutralized SARS-CoV-2 by at least 90% were further tested in duplicate at serial two-fold dilutions to determine 90% endpoint titers. Serum samples were considered seropositive to SARS-CoV-2 when a serum dilution of at least 1:20 reduced no less than 90% of the formation of SARS-CoV-2 viral plaques [15, 24, 25]. Because the potential circulation of other coronaviruses in the region could generate cross-reacting neutralizing antibodies, we used a conservative threshold for detection of neutralizing antibodies of 90%, and we considered seropositive only samples that presented PRNT_90_ titers of 20 or greater.

## Results

All oropharyngeal (n = 96) and rectal (n = 96) swabs were submitted to both real-time RT-PCR protocols and tested negative for SARS-CoV-2 RNA. To investigate previous SARS-CoV-2 exposure, serum samples of all 96 animals were tested by PRNT_90_ for the detection of SARS-CoV-2-neutralizing antibodies. From those, one cat (70S) and one dog (64S), presented PRNT_90_ titer of 80 and 40, respectively. Both individuals were stray animals. They were taken to the veterinary clinics by volunteers who capture abandoned animals for neutering and general health care, and then put them up for adoption. None of those animals had respiratory symptoms or fever at the time of sampling. The cat 70S is a female with 3,3kg, no breed defined (NBD), and one year old. The dog 64S is a female with 13,5 kg, NBD and eight years old. Both animals were sampled in the Barra da Tijuca neighborhood, located in the west zone of Rio de Janeiro.

## Discussion

Based on the serological data presented here, we suggest exposure of a stray cat and a stray dog to SARS-CoV-2, during the ongoing COVID-19 epidemic in Rio de Janeiro, Brazil. Our results corroborate the evidence observed in other countries that not only owned pets, but also stray animals are being exposed worldwide [10–16, 25–27].

Infection is likely to occur only when the animal is in close contact with people who are actively shedding virus or in contaminated environments [11, 13, 15]. Both cat 70S and dog 64S may have been exposed to SARS-CoV-2 by three main routes: 1) contact with other infected animals, 2) contact with infected humans, and finally 3) exposure to a contaminated environment. SARS-CoV-2 RNA has already been detected on public surfaces and in the sewage system in large Brazilian cities, pointing out that these environments can be a potential source of infection [28, 29].

In Wuhan, China, a high seroprevalence for SARS-CoV-2 was observed in cats sampled between January and March 2020, mainly due to the high viral transmission in that period [16]. The same high seroprevalence may be observed in other cities, but more studies are needed to draw a global eco-epidemiological picture [11, 30].

Human-animal transmission events are not restricted to domestic animals. Several large felines in the Bronx Zoo in New York, USA developed symptoms of respiratory illness and tested positive for SARS-CoV-2. The source of infection was identified as a zoo employee, who was actively shedding virus [31]. Despite these results, reverse transmission, that is, from pets to human, has never been demonstrated. At least so far, only one episode of probable direct transmission of animals to humans has been reported and involved an outbreak of respiratory disease in farmed minks in the Netherlands [32]. As observed for other respiratory viruses, as influenza, mink replicates SARS-CoV-2 efficiently and are able to transmit the virus, in addition to developing lung lesions similar to those observed in humans. For those reasons, this species has been used as an excellent model for drug and vaccine testing [33].

One of the strengths of this study was the convenience sampling in two veterinary clinics that did not specifically include only pets owned by COVID-19 patients. The inclusion of stray animals has revealed an important aspect for the surveillance of SARS-CoV-2 in urban centers and may be considered as indicator of environment contamination. Animals were sampled between June and August, when the epidemic reached about 100,000 human confirmed cases of COVID-19 in the city of Rio de Janeiro [34]. Another strength of this study is the use of both highly sensitive and highly specific molecular and serological methods for detection of SARS-CoV-2 infection. Molecular methods included two different well-established real-time RT-PCR protocols for SARS-CoV-2 diagnosis. Antibody detection in sera was accomplished by PRNT_90_ that utilized a highly conservative threshold of 90% neutralization, which is considered one of the most specific serological tests for the differentiation of viral infections in convalescent serum samples [35].

Limitations of our study include the lack of sampling in other regions of the city, and the absence of other coronaviruses in the PRNT_90_ as differential diagnosis. The *Coronaviridae* family has different species of coronaviruses, including the feline enteric coronavirus (FECV) and the feline infectious peritonitis coronavirus (FIPV) that infect cats, and the canine coronavirus (CCoV) and the canine respiratory coronavirus (CRCoV) that infect dogs [34]. The potential serological cross-reactivity between SARS-CoV-2 and these other coronaviruses remains poorly investigated. A recent study suggests little or absent cross-reactivity between SARS-CoV-2 and feline infectious peritonitis virus type I or II [16]. However, unless tested by other coronaviruses, cross-reactivity cannot be fully discarded. The circulation of canine and feline coronaviruses in Rio de Janeiro has already been reported [16, 36–38]. Because of that, instead of using a 50% neutralization criterion as it has been reported elsewhere [22, 39], we decided to adopt the most conservative criteria of 90% neutralization for detection of SARS-CoV-2 neutralizing antibodies, even at cost of some false negatives. If a 50% neutralization was used as positivity criteria, cat 70S and dog 64S would present PRNT_50_ titers ≥320, but no other animal would be seropositive.

In conclusion, the results presented here suggest that stray animals were not currently infected, but may have been exposed to SARS-CoV-2 during the ongoing COVID-19 epidemic in Rio de Janeiro, Brazil. Present findings are in agreement with previous investigations that suggest human-animal transmission of SARS-CoV-2 [9–16]. For that reason, the investigation of SARS-CoV-2 in animal populations by a One Health approach is necessary and should be encouraged [40]. It is important to emphasize that, based on current data, COVID-19 patients must maintain preventive measures of social isolation and pets, such as cats and dogs, should be included in this care. Further studies are needed to establish the role of pets and other animals in the ongoing COVID-19 pandemic. We reinforce that any attempt to abandon or mistreat animals is condemnable and is not justified.

## Supporting information

S1 Database (XLSX)
